# Deep proteogenomic characterization of pancreatic solid pseudopapillary neoplasm reveals unique features distinct from other pancreatic tumors

**DOI:** 10.1186/s40364-025-00875-y

**Published:** 2025-11-27

**Authors:** Atsushi Tanaka, Yusuke Otani, David S. Klimstra, Olca Basturk, Monika M. Vyas, Julia Y. Wang, Michael H. A. Roehrl

**Affiliations:** 1 Department of Pathology, Beth Israel Deaconess Medical Center, Boston, MA USA; 2 Harvard Medical School, Boston, MA USA; 3 Broad Institute of MIT and Harvard, Cambridge, MA USA; 4 Department of Pathology and Laboratory Medicine, Memorial Sloan Kettering Cancer Center, New York, NY USA; 5 Department of Pathology, Yale University School of Medicine, New Haven, CT USA; 6 Curandis, Boston, MA USA; 7 Wyss Institute at Harvard, Boston, MA USA

**Keywords:** Solid pseudopapillary neoplasm, Proteomics, Metabolic adaptation, Protein biomarkers, Tumor immune microenvironment, Therapeutic targets

## Abstract

Solid pseudopapillary neoplasm (SPN) of the pancreas is a rare but distinct disease that remains poorly understood, especially at proteome level. We report comprehensive mass spectrometry-based proteomic analyses of SPN (*n* = 13) and characterize differences from other pancreatic neoplasms, pancreatic ductal adenocarcinoma (*n* = 11) and neuroendocrine tumor (*n* = 10). We discovered that the SPN proteome is uniquely distinct from that of other pancreatic neoplasms. Lysosome-related proteins are enriched and upstream lysosomal processes transcriptional regulators, MITF and TFE3, are overexpressed in SPN. MITF protein expression is more specific for SPN than TFE3, previously considered the most specific immunohistochemical marker. Since lysosomal-related processes are connected to biological energy generation processes, we profiled metabolic pathways and found that SPN is characterized by higher fatty acid oxidation and lower glycolysis than PDAC and high proteasome pathway activity with many proteasomal proteins upregulated, suggesting a possible link to metabolic adaptation mechanisms in low-nutrient environments. Proteomics characterizes SPN as an immune-cold tumor with low MHC class I expression. Proteome-based receptor tyrosine kinase (RTK) pathway profiling suggests PDGFRA and ERBB2 (HER2) as potential candidates for targeted therapy. Our results provide unique proteomic contribution to the understanding of SPN biology and highlight differences from other pancreatic tumors.

**Graphical abstract:**

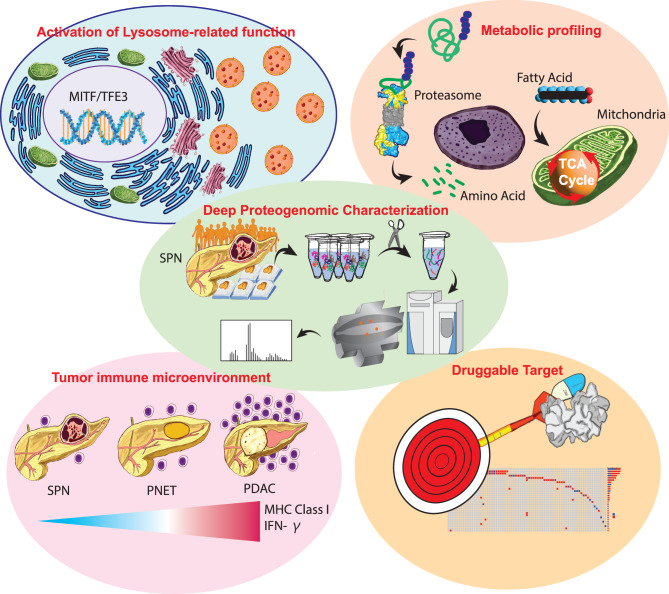

**Supplementary information:**

The online version contains supplementary material available at 10.1186/s40364-025-00875-y.

## Background

Solid pseudopapillary neoplasm (SPN) is a rare neoplasm of the pancreas of unclear histogenesis, and its clinical behavior is distinct from other pancreatic malignant tumors such as pancreatic ductal adenocarcinoma (PDAC). SPN accounts for 1–2% of pancreatic tumors and has a female predominance [1–4]. It predominantly affects young women, typically in the second to fourth decades of life, and often presents as a large, well-circumscribed mass with both solid and cystic components. Solid pseudopapillary neoplasms (SPNs) are typically classified as low-grade malignant tumors, as the majority of patients experience a favorable prognosis following surgical resection. Nonetheless, a subset of these cases may present with aggressive characteristics, including vascular invasion, local recurrence, or distant metastasis [5]. SPNs are histologically heterogeneous tumors characterized by a mixture of solid and pseudopapillary architecture. Solid areas consist of uniform tumor cells and delicate capillary vasculature, while pseudopapillae form due to detachment of tumor cells from fibrovascular cores. The stroma often shows hyalinization, hemorrhage, and degenerative changes such as foamy macrophages and calcifications.

Genomic studies have revealed that most SPNs harbor somatic mutations in *CTNNB1*, unlike the much more common PDAC (that harbors alterations in *KRAS*, *TP53*, *CDKN2A*, and *SMAD4*) [6–8]. Studies using immunohistochemistry have demonstrated nuclear β-catenin accumulation in SPN [8, 9], but this alteration is not specific for SPN as it can be seen in other tumor types including pancreatic acinar cell carcinoma [10], pancreatoblastoma [11], colon cancer [12], desmoid tumor [13], solitary fibrous tumor [14], or gastric cancer [15]. Very few genomic and transcriptomic analyses of SPN have been performed [8, 16, 17], and there is no comprehensive information on the proteomic characteristics of SPN beyond a few older studies performed at low proteome depth and with very few patient samples [18, 19]. In addition, the tumor immune microenvironment of SPN has not been investigated at the protein level. Since immunotherapy has emerged as a new treatment modality and shows effectiveness in many different cancers [20], investigating the tumor immune microenvironment of SPN may be beneficial for developing new therapeutic options.

To address these gaps, we performed the first comprehensive comparative deep proteomic analysis of SPN and two other major pancreatic tumor types, PDAC and pancreatic neuroendocrine tumor (PanNET). Our study characterizes SPN-specific protein biomarkers, signal pathway enrichment, tumor immune microenvironment, and possible therapeutic targets.

## Materials and methods

### Clinical specimens and data

We obtained formalin-fixed, paraffin-embedded (FFPE) tissues from a total of 13 SPNs, 11 PDACs, 10 PanNETs (defined as well-differentiated neoplasms, with 5 classified as G1 and 5 as G2 tumors), and 9 benign exocrine pancreatic tissues (cohort 1) [21]. We also generated a validation IHC cohort comprised of 62 PDACs, 172 PanNETs (88 G1, 84 G2), and 51 SPNs (cohort 2). All cases were reviewed by pathologists. Clinical data, including patient demographics and targeted sequencing results were retrieved from medical records. Information regarding the histological type and other pathological factors was retrieved from diagnostic reports. Additionally, the tumor purity for each sample was checked by pathologists. MSKCC and BIDMC Institutional Review Boards (16–1683, 2023P000933) approved this study.

### Targeted DNA sequencing

Processed whole-exome sequencing data, including SNV-INDEL calls and copy number calls of SPN in the cohort 1, were obtained from the authors of a previous report [8]. MSK-IMPACT targeted gene panel sequencing of PanNET and PDAC samples in the cohort 1 was conducted as previously described [22]. Ten unstained sections, each 10 micrometers thick, were obtained from both the tumor and normal FFPE blocks. To increase the tumor content, the tumor regions were dissected macroscopically, and DNA was extracted by the DNeasy Tissue kit, followed by fragmentation with a Covaris E200 (Qiagen). DNA libraries were created following the KAPA HTP protocol. These pooled libraries underwent sequencing on the Illumina HiSeq 2500 platform (over 500× median). Genomic alterations, including substitutions, insertions and deletions (indels), copy number variations, and rearrangements, were identified and analyzed in comparison to the patient’s corresponding normal sample. All tests were conducted in a laboratory compliant with CLIA.

### Tissue proteome extraction and MS sample preparation from FFPE tissue

Sample preparation from the cohort 1 FFPE blocks for mass spectrometry analysis was conducted according to a previous proteome analysis protocol for FFPE tissue developed in our laboratory [21, 23, 24]. Ten unstained 10-micrometer sections were obtained from FFPE tissues, while adjacent 4-micrometer sections underwent H&E staining to verify tumor content. Tumor regions were dissected manually from slides. After dewaxing, a lysis buffer of 100 mM Tris and 5% SDS was added, followed by sonication and incubation at 98 °C for 20 minutes at 1000 rpm, and at 80 °C for 2 hours at 1000 rpm. After centrifuging at 14,000 g for 30 minutes at 4 °C, we collected the supernatant with soluble proteins. Using BCA assay (Pierce), we determined protein concentrations. Proteomes (100 µg per sample) underwent double digestion using Trypsin/Lys-C Mix (Promega), and were processed with S-Trap column (ProtiFi). Samples were cleaned on a C18 StageTip, dried with a SpeedVac machine, mixed with 3% acetonitrile and 0.1% formic acid, and kept at −80 °C until they were ready for mass spectrometry analysis.

### Proteomics of FFPE tissue by liquid chromatography-mass spectrometry (LC-MS)

Desalted peptides in 3% acetonitrile/0.1% formic acid were injected into a C18 capillary column (Peptide BEH 1.7 μm × 75 μm × 250 mm, Waters) coupled to a Q Exactive Plus mass spectrometer (Thermo Scientific) via a nano ACQUITY UPLC system (Waters). Peptides were eluted using a non-linear gradient over 200 minutes, from 2% to 35% buffer B (0.1% formic acid and pure acetonitrile) at 300 nl/min flow rate, followed by a 5-minute column wash with 90% buffer B and re-equilibration with 98% buffer A (0.1% formic acid in HPLC-grade water). Mass spectrometry data were collected by switching between full scans and 10 data-dependent MS/MS scans using TopN method. The target was 1 × 10^6^ ions within 380–1,600 m/z range, with 50 ms maximum injection time and 70,000 resolution at 200 m/z in profile mode. Precursors were chosen with 1.5 m/z isolation width and fragmented using HCD at 27 eV normalized collision energy. MS/MS scans were recorded at 17,500 resolution at 200 m/z, with 5 × 10^4^ ion target value, 50 ms maximum injection time, 15 seconds dynamic exclusion, in centroid mode.

### Protein identification, quantification, and differential expression analysis

We processed LC-MS raw data files by using Proteome Discoverer (Thermo Scientific, version 3.0.1.27). Raw files were searched using the CHIMERYS search engine with a Homo sapiens UniProt protein database downloaded on 2022/10/27 (42,313 entries). Methionine oxidation was set as a variable modification, whereas cysteine carbamidomethylation was specified as a fixed modification. Up to two missed cleavages by trypsin were allowed. To manage the peptide false discovery rate (FDR), a reversed sequence decoy approach was employed, with a 1% FDR set as the identification threshold. Protein quantification was conducted utilizing unique and razor peptides in the PD software. Precursor abundance was determined based on intensity, while peptide abundance normalization was performed according to the total peptide amount of each sample. Normalized protein abundance was calculated by aggregating the abundances of the associated peptide groups in the PD software. We kept proteins with expression values present in more than 50% of all samples. Missing values were imputed using the K-nearest neighbor (KNN) imputation (the impute R package) [25] with k = 10 based on similarity across all samples. Default values for other parameters in imputation step were used. To identify differentially expressed proteins between tumor types and normal pancreas, we used the edgeR package (v3.30.3) [26] in R (v4.3.2) [26] for normalized protein abundance data analysis. Sample groups were defined based on histological types. The group variable was treated as a factor with a specified order to control contrast directions. We constructed a DGEList object using the DGEList() function and specified the design matrix using model.matrix() function of the edgeR. Dispersion estimates were computed with estimateDisp() to model biological variability across samples. Differential expression tests were performed using exactTest() between histological groups. For each comparison, we extracted log2 fold changes, exact test p-values. The Benjamini-Hochberg method was employed for multiple testing correction to manage the false discovery rate (FDR). Proteins with FDR < 0.05 were considered significantly differentially expressed.

### Proteomic pathway enrichment analyses

The Metascape web application was used to conduct pathway enrichment analyses with default settings [27].

### Single sample gene set enrichment analysis (ssGSEA) based on proteomic data

To functionally characterize the proteomic profile of each individual sample, we performed single-sample Gene Set Enrichment Analysis (ssGSEA). This approach computes an enrichment score for each gene set per sample, enabling pathway-level interpretation of proteomic activity in a sample-specific manner. We used the ssGSEA 2.0 implementation available from the Broad Institute GitHub repository (https://github.com/broadinstitute/ssGSEA2.0) to compute scores. As input, we provided the normalized protein abundance matrix, where rows corresponded to gene symbols and columns to individual samples. We projected the protein expression matrix onto multiple gene set collections, including Gene Ontology (GO) biological process term, Hallmark gene sets, KEGG pathways, and Reactome pathways. All gene sets were obtained from the Molecular Signatures Database (MSigDB; version 7.4). Entries with fewer than 3 overlapping proteins in a given gene set were excluded from that calculation. The following ssGSEA parameters were used: statistic = “area.under.RES”, sample.norm.type = “log,” weight = 0.75, nperm = 1000, min.overlap = 3, output.score.type = “NES”, correl.type = “z.score” [28]. The resulting NES matrix (samples as columns, gene sets as rows) was used for downstream analyses such as pathway comparisons between tumor subtypes.

### Receptor tyrosine kinase pathway enrichment analysis

We selected all receptor tyrosine kinase-related pathways from the KEGG and Reactome databases that had proteome-based ssGSEA scores from both benign tissue and SPN. We calculated average score differences between benign tissue and SPN. We used the Wilcoxon rank-sum test to statistically evaluate score differences.

### Tissue microarray (TMA) construction

To facilitate IHC assessment, we generated TMAs from FFPE blocks based on our laboratory’s protocol [29–35]. We also added acinar cell carcinoma (*n* = 8) and pancreatoblastoma (*n* = 3) cases to the cohort 1 samples to expand the breadth of entities represented on the TMA. Three distinct 2-mm tissue cores were extracted from donor paraffin block and placed into tissue array blocks by the TMA Grand Master arrayer (3DHistech). The target areas were chosen after a thorough examination of the histological slides for each donor block.

### Immunohistochemistry (IHC)

Sections of FFPE tissues, each 4 µm thick, were prepared. Xylene was used to remove the paraffin. We retrieved antigens through heat-mediated epitope retrieval at pH 6.0. We performed immunohistochemical staining with a Leica BOND-MAX automated system. The antibodies used were anti-MITF (Abcam, ab303530, 1/2,000 dilution), anti-TFEB (Abcam, ab267351, 1/1,000 dilution), anti-TFE3 (Abcam, ab179804, 1/4,000 dilution), anti-CD3 (Leica Microsystems, NCL-L-CD3-565, 1/200 dilution), anti-CD20 (Abcam, ab64088, 1/200 dilution), anti-CD163 (Abcam, ab182422, 1/500 dilution), and anti-HLA-ABC (Abcam, ab225636, 1/4,000 dilution). In addition, receptor tyrosine kinases were assessed by using anti-PDGFRA (Cell Signaling Technology, #3174, 1/200 dilution), anti-PDGFRB (Cell Signaling Technology, #4564, 1/100 dilution), anti-HER2 (Cell Signaling Technology, #4290, 1/100 dilution), anti-ERBB4 (Abcam, ab137412, 1/800 dilution), anti-FGFR1 (Proteintech, #60325–1-IG, 1/1500 dilution), anti-FGFR2 (Cell Signaling Technology, #23328, 1/100 dilution), anti-FGFR3 (Invitrogen, #MA5-38521, 1/400 dilution), and anti-FGFR4 (Proteintech, #11098–1-AP, 1/750 dilution). We assessed IHC results of MITF, TFEB, TFE3, and HLA-ABC in a semiquantitative manner. The intensity of staining in individual tumor cells was rated on a scale of 0, 1+, 2+, or 3+, with the average taken from three separate tissue cores per case. To calculate the total weighted IHC score (IHC H-score) for a sample, the expression intensity of specific tumor regions (scored from 0 to 3+) was multiplied by their relative contribution (0–100%) to the overall tumor area, and these values were summed to produce a total weighted score. Consequently, IHC H-scores can theoretically range from 0 to 300. IHC for receptor tyrosine kinases typically exhibited uniform staining and was classified as negative, weakly stained, or strongly stained. Two pathologists independently evaluated all tissue samples. If there were any differences in the immunohistochemical evaluations between the two evaluators, they reviewed the cases jointly to reach a consensus score. For CD3, CD20, and CD163 IHC, we counted positive cells per tissue core.

### Analysis of receiver operating characteristic (ROC) and area under the curve (AUC)

To evaluate the biomarker performance of IHC markers (MITF and TFE3), we conducted ROC curve analysis (pROC package, version 1.18.5) [36]. ROC curves were generated for each marker by plotting sensitivity (true positive rate) against 1-specificity (false positive rate) across a range of thresholds. The area under the ROC curve (AUC) was calculated as a summary measure of diagnostic accuracy. An AUC of 1.0 indicates perfect discrimination, whereas an AUC of 0.5 corresponds to random classification. ROC curves were visualized using the ggroc function, with diagonal reference lines indicating random classification performance.

### Proteome-based immune score calculation

In order to determine immune infiltration levels across tumor samples, we utilized the ESTIMATE algorithm through the ESTIMATE R package (v1.0.13) [37], which was applied to the normalized protein expression matrix derived from all samples. The analysis was conducted using the estimateScore() function with default parameters, which computes immune scores based on gene expression signatures associated with immune cell infiltration. The resulting immune scores were used for comparative analysis between tumor types [21, 23, 38].

### Proteome-based cell type deconvolution

To infer the relative abundance of immune cell types from the proteomics data, we performed cell-type deconvolution analysis using the xCell algorithm (xCell R package, v1.1.0) [39]. xCell is a gene signature-based methodology that calculates enrichment scores for various immune cell types utilizing expression profiles. We provided the protein expression matrix (expression level ranking) of this study into xCell. This approach emphasizes the relative expression of proteins within each sample and enables xCell to compute enrichment scores despite differences in measurement platform. All analyses were performed using the default parameters of the xCell pipeline, without modification of gene signatures or score scaling. The resulting enrichment scores for each immune cell type were used to compare cell-type composition across SPN, PDAC, and PanNET samples.

### Dimensionality reduction using PCA and UMAP

To evaluate global variations in protein expression among the sample groups, we conducted principal component analysis (PCA) and uniform manifold approximation and projection (UMAP), focusing exclusively on proteins quantified across all samples without any missing values. We performed PCA with the prcomp() function in R and performed UMAP with the umap package (version 0.2.10.0) (with n_neighbors = 16) (https://github.com/tkonopka/umap) on the same expression matrix.

### Statistical analyses

All statistical analyses were performed using R (version 4.3.2) [40]. Numerical values were analyzed by the Wilcoxon rank-sum test. If we used other statistical test methods, we noted it in the description.

## Results

### SPN proteomes are distinct from other tumor types and benign tissue

**Fig. 1:**
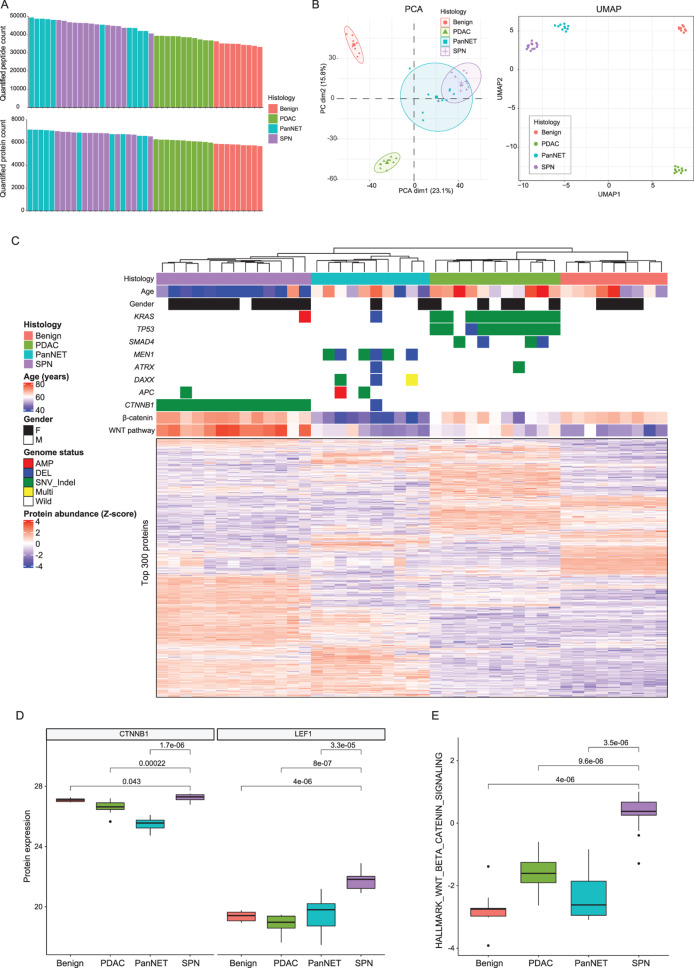
Proteogenomic landscape of SPN. (**A**) Bar plots of quantified peptide counts and protein counts. The mean quantified peptide counts are as follows: 34,643 for benign, 38,349 for PDAC, 45,964 for PanNET, and 45,507 for SPN. Similarly, the mean quantified protein counts are 5,799 for benign, 6,155 for PDAC, 6,897 for PanNET, and 6,811 for SPN. (**B**) Proteomic profiles from benign pancreas, PDAC, PanNET, and SPN samples are analyzed using principal component analysis (PCA, left) and uniform manifold approximation and projection (UMAP, right). Each point represents one sample, colored by histologic group. Ellipses indicate 95% confidence intervals. Only proteins quantified by LC-MS across all samples were utilized for PCA and UMAP analysis. (**C**) Summary protein expression heatmap with genomic alterations and demographic features. The top 300 proteins (whose mean absolute deviations across all samples falls within the top 300 of all quantified proteins) are shown. Unsupervised clustering of rows and columns was applied. In the heatmap, the proteome of each histology shows a clearly unique protein expression profile. Genome status: AMP, amplification; DEL, deletion; SNV_Indel, single nucleotide variant, small insertion, and/or small deletion; multi, multiple events of AMP, DEL, SNV_Indel; wild, no genomic alteration. (**D**) The mass spectrometry-based protein expression levels of CTNNB1 (β-catenin) and LEF1 are significantly elevated in SPN compared to other groups. Wilcoxon rank-sum tests between groups were used as indicated by brackets. Horizontal lines represent 25^th^, 50^th^, and 75^th^ percentiles from bottom to top, respectively. Whiskers indicate 1.5×IQR. Protein expression levels are shown on a log2-transformed scale. (**E**) Hallmark WNT/β-catenin pathway activity of each sample, calculated by ssGSEA on the normalized proteomic matrix using the MSigDB hallmark WNT/β-catenin gene set. The activity score of the WNT/β-catenin pathway in SPN demonstrates significantly higher activity compared to other groups. Wilcoxon rank-sum tests between groups were used as indicated by brackets. Horizontal lines represent 25^th^, 50^th^, and 75^th^ percentiles from bottom to top, respectively. Whiskers indicate 1.5×IQR

We collected FFPE samples from 13 SPNs, 11 PDACs, 10 PanNETs, and 9 benign exocrine pancreatic tissues (Table S1). As expected, the SPN group exhibited the lowest mean age and a predominance of female patients in comparison to the PDAC and PanNET groups [41]. LC-MS proteomic analysis quantified 117,632 peptides and 10,670 proteins in total, and 6,497 proteins were quantified in over 50% of the cohort samples. The quantified peptides in each sample ranged from 33,189 to 49,321, with a mean of 41,508 and a median of 41,908. Similarly, the quantified proteins in each sample ranged from 5,678 to 7,129, with a mean of 6,451 and a median of 6,574 (Fig. 1A). PCA and UMAP of the proteomic profiles revealed distinct clustering of each histologic group (Fig. 1B). While PCA showed partial overlap between SPN and PanNET, UMAP demonstrated clearer separation, highlighting the unique proteomic signature of SPN relative to other pancreatic tumors and benign tissue. Unsupervised clustering of the protein heatmap based on Euclidean distance also showed that the SPN proteome was clearly different from the others (Fig. 1C). Gene alterations in *MEN1*, *ATRX*, and *DAXX* were enriched in the PanNET group [42]. In the PDAC group, mutations in *KRAS*, *TP53*, and *SMAD4* were enriched [43]. Gene mutations in *APC* and *CTNNB1* (WNT/β-catenin pathway) were enriched in SPN [7]. Concordant with genomic alterations, the average protein expression of β-catenin and LEF1 (one of β-catenin’s cofactors) was highest in SPN (Fig. 1D), resulting in the highest activation of the WNT/β-catenin pathway (Fig. 1E). Interestingly, unbiased clustering grouped all samples with the same pathological diagnosis together, suggesting that proteome profiling alone (even without genomic or any other information) has diagnostic power. This observation is potentially important because these tumors sometimes mimic each other morphologically [44]. We hypothesized that disease-specific protein biomarkers exist and may provide a powerful means of differentiating these diseases.

### Proteome profiling unveils specific protein markers of SPN and activated protein module networks

**Fig. 2:**
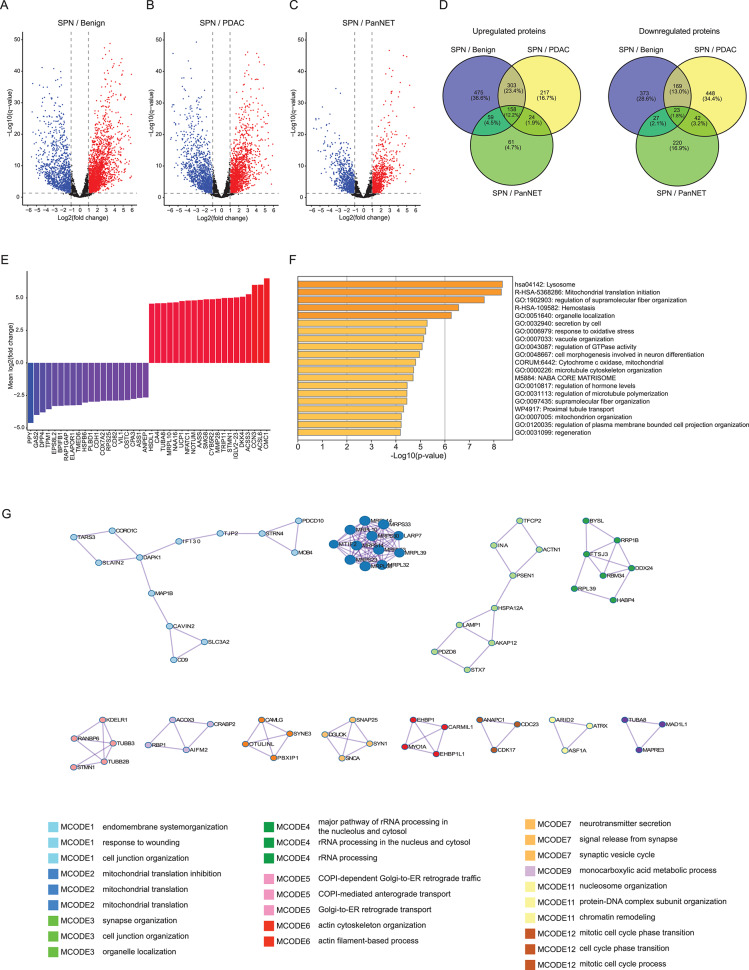
Specific protein markers of SPN and characteristic proteomic modules. (**A**) Volcano plot of differential protein expression (SPN vs. benign pancreatic tissue). Red and blue dots represent significantly upregulated and downregulated proteins in SPN, respectively. (**B**) The volcano plot illustrates the differential protein expression between SPN and PDAC. Red dots indicate proteins that are significantly upregulated in SPN, while blue dots denote those that are significantly downregulated. (**C**) The volcano plot illustrates the differential protein expression between SPN and PanNET. Red dots indicate proteins that are significantly upregulated in SPN, while blue dots denote those that are significantly downregulated. (**D**) Venn diagrams of upregulated and downregulated proteins. Among the significantly expressed proteins (q-values <0.05) from all three pairwise comparisons (**A**–**C**), only upregulated or downregulated proteins with absolute log2(fold change) values >2 were included. 158 proteins were consistently upregulated and 23 proteins were consistently downregulated in SPN relative to PDAC, PanNET, and benign tissue. Thus, a total of 181 proteins meet the criteria of SPN-specific protein candidates. (**E**) Of the 181 SPN-specific proteins in (**D**), the top 20 downregulated and top 20 upregulated proteins are shown ranked by average log2(fold change) across the three pairwise comparisons. (**F**) Metascape pathway enrichment analysis based on 370 highly upregulated proteins in SPN relative to benign pancreatic tissue (log2(fold change)>3). The top 20 enriched gene set terms are shown. (**G**) MCODE analysis, which is an automated molecular complex detection algorithm based on a knowledge database of protein-protein interactions, of 370 highly upregulated proteins in SPN relative to benign pancreatic tissue (log2(fold change)>3). Twelve unique protein modules were identified and are shown as interaction networks. Among the 12 modules, 10 exhibit a significant enrichment of GOBP terms within their modules as shown. The (up to three) top enriched GOBP terms for each of the 10 MCODE modules are listed. Small vesicle-related terms are enriched in several protein modules

We performed pairwise differential proteome analyses between benign pancreatic tissue and SPN, PDAC and SPN, and PanNET and SPN. In the analyses, 3,456 (2,273 upregulated, 1,183 downregulated), 3,085 (1,718 upregulated, 1,367 downregulated), and 1,931 (959 upregulated, 972 downregulated) significantly dysregulated proteins ( > 2-fold change) were found in these comparisons, respectively (Fig. 2A, B, C, Table S2). To identify SPN-specific proteins, we investigated which proteins were shared versus not shared in these differential analyses. Among the proteins whose absolute fold-change values were > 4 (i.e., abs (log2(fold change)) > 2) with q-values < 0.05, 181 proteins (158 upregulated, 23 downregulated) showed significant expression differences in all pairwise comparisons, suggesting SPN specificity (Fig. 2D, Table S3). Although LEF1, a known protein marker of SPN [45], was highly expressed in SPN compared to the other entities (Fig. 1D), LEF1 was not included in Table S3 because of its lower fold-change value than the inclusion threshold (log2 (fold change) values were 1.5 (SPN/benign), 1.9 (SPN/PanNET), and 2.2 (SPN/PDAC)). The top 20 upregulated and top 20 downregulated SPN-specific proteins are shown in Fig. 2E. These proteins may be good candidates as SPN-specific proteins for clinical diagnostic use. For example, ACSL6 (2^nd^ most upregulated protein) and ACSS3 (4^th^ most upregulated) are both acyl-CoA synthetases, which contribute to various biological processes including ATP generation through fatty acid oxidation pathway [46]. This function may indicate metabolic adaptation mechanism of SPN (see next section).

To elucidate significantly enriched protein-protein interaction networks of SPN, we conducted pathway enrichment analyses using Metascape [27] based on 370 highly upregulated proteins relative to benign pancreatic tissue (log2 (fold change) > 3). Among the top 20 enriched gene set terms, we found that many terms were related to lysosomal function or small vesicle organization, such as “lysosome”, “secretion by cell” or “vacuole organization” (Fig. 2F). Using MCODE analysis implemented in Metascape [47], we then searched protein module networks of SPN based on protein-protein interactions of the 370 highly upregulated proteins, resulting in 12 protein modules (Fig. 2G). Over representative analysis of GO terms for proteins in each module revealed that several modules were related to vesicle organization such as “endomembrane system organization”, “organelle localization”, or “synaptic vesicle cycle”.

### MITF as a specific protein marker and its possible role in the metabolic adaptation of SPN under nutrient deprivation

**Fig. 3:**
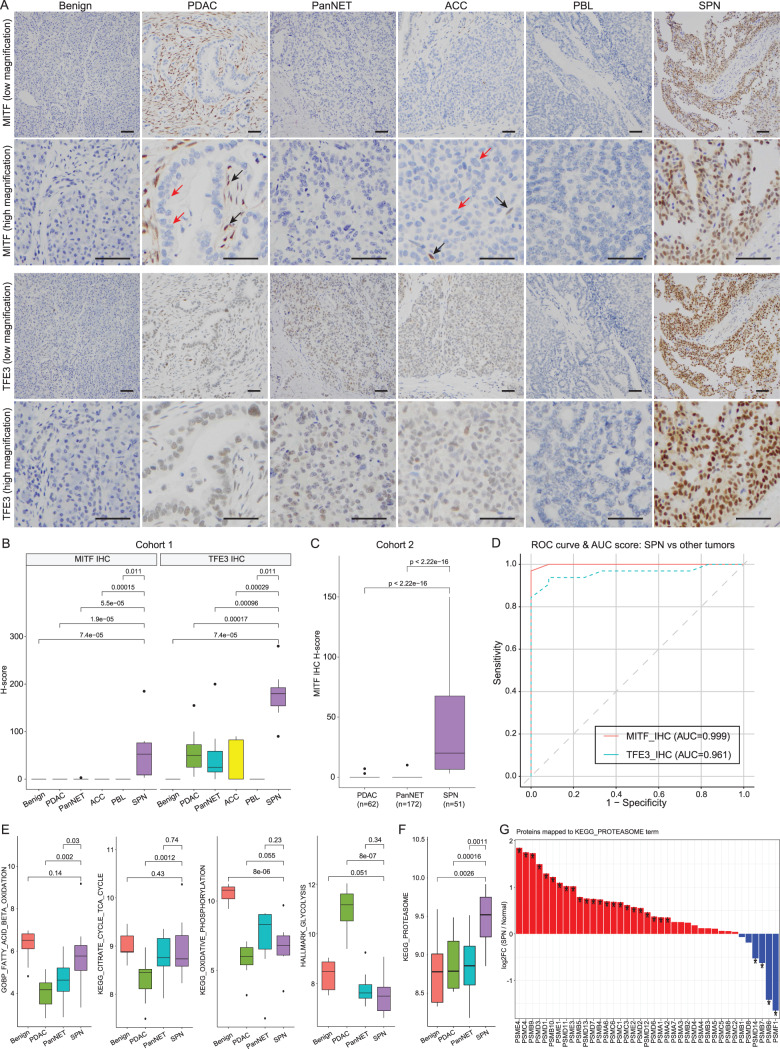
Metabolic adaptation of SPN. (**A**) Representative IHC images of MITF and TFE3. SPN shows strong nuclear positivity for both MITF and TFE3 in tumor cells. By contrast, benign pancreatic tissue, PDAC, PanNET, ACC, and PBL show no nuclear staining for MITF. While TFE3 is negative in benign pancreatic tissue, PDAC, PanNET, and ACC show low-to-moderate nuclear positivity for TFE3 in tumor cells. Tumor cells of PDAC and ACC show no positivity for MITF (red arrows), but some stromal cells show positivity for MITF (black arrows). Bars, 50 µm. (**B**) IHC score plots of MITF and TFE3 expression. IHC of 9 benign, 11 PDAC, 10 PanNET, 8 ACC, 3 PBL, and 13 SPN samples was assessed. SPN shows significantly higher expression of TFE3 and MITF than the other subtypes. In addition, MITF shows exclusive specificity for SPN, which is in contrast to the TFE3 staining pattern. Wilcoxon rank-sum tests between groups were used as indicated by brackets. Horizontal lines represent 25th, 50th, and 75th percentiles from bottom to top, respectively. Whiskers indicate 1.5×IQR. (**C**) IHC score plots of MITF expression by using the validation cohort. IHC of 62 PDAC, 172 PanNET, and 51 SPN samples was assessed. SPN shows significantly higher MITF expression than the other subtypes. Wilcoxon rank-sum tests between groups were used as indicated by brackets. Horizontal lines represent 25^th^, 50^th^, and 75^th^ percentiles from bottom to top, respectively. Whiskers indicate 1.5×IQR. (**D**) Receiver operating characteristic (ROC) curves comparing the diagnostic performance of MITF and TFE3 IHC for distinguishing SPN from other pancreatic tumors. Only cohort 1 data are used. The red curve corresponds to MITF IHC (area under the curve (AUC) = 0.999; best threshold = 1.50; sensitivity = 0.97; specificity = 1.00; positive predictive value (PPV) = 1.00; negative predictive value (NPV) = 0.92), and the blue dashed curve corresponds to TFE3 IHC (AUC = 0.961; best threshold = 120.00; sensitivity = 0.94; specificity = 0.92; PPV = 0.97; NPV = 0.85). The diagonal gray dashed line indicates the reference line for random classification (AUC = 0.5). (**E**) ssGSEA score plots of key metabolic processes. Wilcoxon rank-sum tests between groups were used as indicated by brackets. Horizontal lines represent 25^th^, 50^th^, and 75^th^ percentiles from bottom to top, respectively. Whiskers indicate 1.5×IQR. (**F**) ssGSEA score plot of the KEGG proteasome term. Wilcoxon rank-sum tests between groups were used as indicated by brackets. Horizontal lines represent 25^th^, 50^th^, and 75^th^ percentiles from bottom to top, respectively. Whiskers indicate 1.5×IQR. (**G**) Protein expression changes of members of KEGG proteasome term between SPN and benign tissue. * indicates a q-value <0.05

Our differential expression analysis identified 158 proteins significantly upregulated in SPN compared to benign, PDAC, and PanNET tissues, including several potentially useful diagnostic candidates such as DKK4. However, rather than focusing on individual top-ranked proteins, our aim was to identify upstream regulatory factors that may act as central drivers of SPN biology and serve as robust, specific biomarkers. Pathway enrichment analysis and protein-protein interaction network analysis revealed that many of the upregulated proteins in SPN were associated with lysosomal and vesicle-related processes (Fig. 2E, F, G). Since these pathways are transcriptionally regulated by the MITF/TFE family via CLEAR (Coordinated Lysosomal Expression and Regulation) elements [48], we hypothesized that this family, specifically MITF, TFE3, and TFEB, may be overexpressed in SPN and represent key regulatory features distinguishing SPN from other pancreatic tumors. Given that TFE3 has been previously reported as a marker of SPN, we investigated whether MITF/TFEB might offer improved diagnostic specificity, and whether its expression could reflect broader biological processes relevant to SPNs metabolic phenotype. We therefore investigated the expression of MITF, TFE3, and TFEB by immunohistochemistry (IHC) across pancreatic tumor types. Although TFEB was not detectable in SPN (data not shown), TFE3 and MITF were both highly expressed in SPN compared to other tissue types, including tumor and benign tissues (Fig. 3A, B, C, cohorts 1/2). In addition, TFE3 also showed weak expression in PDAC, PanNET, and ACC. In contrast, MITF showed essentially no expression in these tumor types in cohort 1. Independent validation in cohort 2 confirmed that MITF is specific for SPN (Fig. 3C). Using cohort 1 MITF/TFE3 IHC results, we then conducted a receiver operator characteristics (ROC) analysis (Fig. 3D). The area under the curve (AUC) score of MITF was higher than that of TFE3. These results suggest MITF could be a useful protein marker of SPN in addition to the SPN-specific proteins shown above (Fig. 2D, E, Table S3).

IHC staining showed that expression of TFE3 in SPN was predominantly nuclear, suggesting that SPN may be under nutrient starvation [49, 50]. To survive nutrient deprivation, metabolic adaptation is required. Proteome-based metabolic pathway analyses revealed differences between SPN, PDAC, and PanNET (Fig. 3E). In general, SPN showed a benign-like metabolic profile compared to other tumors. This clearly contrasts with the metabolic processes of PDAC, a highly aggressive tumor with a high proliferation rate (high energy consumption). In PDAC, glycolysis was strongly activated, while the TCA cycle was downregulated, consistent with the known Warburg effect in PDAC [51]. We also studied proteasome pathway activation as a feature of nutrient deprivation [52]. The KEGG proteasome pathway score of proteasomes was the highest for SPN (Fig. 3F). Many proteasomal proteins were significantly upregulated relative to the benign tissues (Fig. 3G).

### SPN has an immune cold microenvironment compared with PDAC

**Fig. 4:**
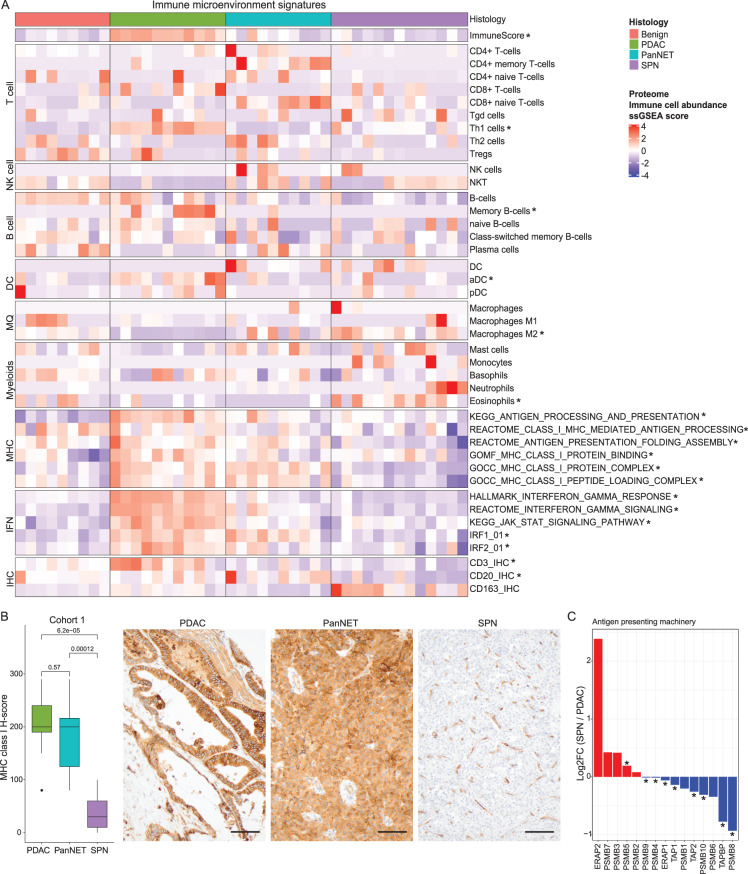
Immune microenvironment profiling of SPN. (**A**) heatmap of inferred immune cell abundances and ssGSEA scores of the MHC-related and IFNγ pathways. CD3, CD20, CD163-positive cell count heatmap by IHC is also shown at the bottom of the heatmap. * indicates statistical significance between PDAC and SPN (Wilcoxon test, *p* < 0.05). (**B**) Boxplot of MHC class I H-score between PDAC, PanNET, and SPN with representative IHC images. Bars, 100 μm. (**C**) Protein expression changes of the antigen presenting machinery between SPN and PDAC. * indicates statistical significance (q-value < 0.05)

To characterize tumor immune microenvironments, we deconvoluted the immune cell composition using xCell. SPN generally harbored relatively low T/B-cell infiltration, moderate NKT cell infiltration, high M2-type macrophage infiltration, and myeloid infiltration. PDAC showed the highest activation of the MHC class I and IFNγ pathways among the three tumors. In contrast, SPN showed the lowest activation of the MHC class I and IFNγ pathways. The immune score calculated by ESTIMATE showed that SPN and PanNET had low scores compared with PDAC, classifying SPN as immune cold and PDAC as immune hot. To verify these characteristics, we conducted IHC assessments of CD3 (a T cell marker), CD20 (a B cell marker), CD163 (M2-type macrophage marker), and MHC-class I (HLA-A/B/C) (Fig. 4A). Consistent with our findings, CD3/20-positive T/B cell counts were higher in PDAC compared to SPN. In contrast, CD163-positive macrophages were more prevalent in SPN. In alignment with immune pathway analyses, IHC results demonstrated a significant downregulation of MHC class I in SPN compared to PDAC and PanNET, suggesting potential mechanisms by which SPN may evade host immune surveillance (Fig. 4B). To further explain differences between immune cold and hot states, we investigated expression level differences in antigen processing machinery (APM) components of SPN versus PDAC (Fig. 4C). Among the 15 APM proteins, most were significantly downregulated in SPN compared to PDAC. In SPN, the IFNγ pathway, which triggers the JAK/STAT pathway and IRF transcription while promoting MHC class I-related proteins, was also found to be less active compared to PDAC (Fig. 4A).

### Proteomic receptor tyrosine kinase (RTK) pathway assessment reveals potential therapeutic targets against SPN

**Fig. 5:**
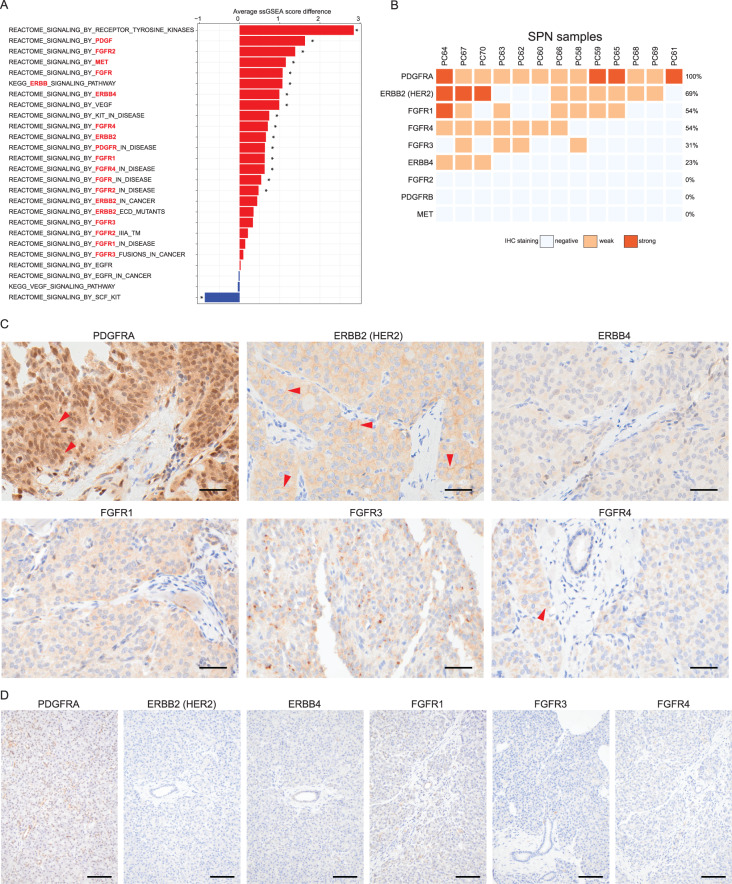
Receptor tyrosine kinase profiling of SPN. (**A**) Activation status of KEGG and Reactome RTK pathways ordered by average ssGSEA score difference (SPN vs. benign tissue). Pathways highlighted in red were also tested by IHC for individual protein expression of the entry point RTKs. * indicates statistical significance (q-value < 0.05). (**B**) Individual protein expression of selected upstream RTKs in SPNs. (**C**) Representative RTK IHC images of SPNs. IHC staining is predominantly cytoplasmic, but PDGFRA, ERBB2 (HER2), and FGFR4 IHC also exhibit focally membranous staining (red arrow heads). Bars, 40 μm. (**D**) Representative RTK IHC images of benign pancreas tissue. Bars, 100 μm

Given that there is no molecularly targeted therapy for SPN and that RTKs play a role in many neoplasms and can be targeted [53–56], we used our proteomic data to rank KEGG and Reactome RTK pathways by their average ssGSEA enrichment score differences between benign tissue and SPN (Fig. 5A). A higher difference score may indicate activation of a pathway relative to benign tissue. Proteome-based RTK pathway analysis suggests activation of pan-RTK, pan-PDGF, pan-FGFR, and pan-ERBB pathways, involving specifically PDGFRA/B, FGFR1/2/3/4, ERBB2/4, and MET pathways (Fig. 5A, highlighted in red). In contrast, KIT and VEGF pathways showed inconclusive results. We then investigated individual RTK protein expression levels by IHC as the upstream entry point of each signaling pathway. We found that more than half of SPNs showed positivity for PDGFRA, ERBB2 (HER2), FGFR1, or FGFR4 proteins (Fig. 5B, C), whereas benign pancreatic tissue showed almost no staining (Fig. 5D). This suggests each of these proteins is therapeutically targetable.

## Discussion

We present the first unbiased deep proteomic study of SPN with comparison to other pancreatic neoplasms (Fig. 1A, B, C). Despite the rarity of SPN, our patient cohort is more than four times larger than the only two other studies that were performed, which had only three patients each and shallow proteome depth [18, 19]. The proteome of SPN is distinct from other entities, and unsupervised clustering correctly classifies the samples into our four sample types (benign, PDAC, PanNET, SPN). Our proteomic data confirm the activation of the WNT/β-catenin pathway that has been well described previously in SPN (Fig. 1D, E) [8, 9].

Our study builds upon and substantially extends previous molecular analyses of SPN, including transcriptomic studies by Selenica et al. [8], Cavard et al. [16] and Park et al. [17], and proteomic analyses by Park et al. [18] and Zhu et al. [19]. Prior transcriptomic investigations identified activation of Wnt/β-catenin, Hedgehog, and androgen receptor signaling pathways in SPN. Our deep proteomic profiling confirms the prominent activation of the Wnt/β-catenin pathway in SPN (Fig. 1D, E). Our proteomic data did not highlight Hedgehog or androgen receptor signaling among the most enriched pathways. These discrepancies may reflect not only differences in sample composition and protein detection depth, but also the poor correlation between mRNA and protein abundance, as has been widely observed in prior large-scale multiomics studies including our report [23, 57]. Differences in post-transcriptional regulation, protein turnover, and technical limitations of mass spectrometry-based detection may also contribute to the observed divergence between transcriptomic and proteomic findings.

Park et al. [18] reported increased expression of glycolysis-related proteins in SPN compared to non-neoplastic pancreatic tissue. In contrast, our data suggest that glycolytic activity in SPN is relatively low, and that SPN instead exhibits a more oxidative metabolic profile, with upregulation of fatty acid β-oxidation and proteasome activity (Fig. 3E, F, G). This difference may reflect variations in sample size, proteome depth, and analytical approach, as our dataset includes a larger number of SPN cases and broader proteomic coverage. Together, these findings support the interpretation that SPN favors oxidative metabolism, consistent with its relatively indolent clinical behavior. Zhu et al. [19] reported enrichment of ER-associated proteins in SPN. While ER-related categories were not among the top pathways in our analysis, we observed significant upregulation of lysosomal and small vesicle-related processes, which may reflect overlapping mechanisms involving intracellular trafficking and protein handling. These findings collectively point to dysregulation of endomembrane systems as a characteristic feature of SPN biology.

We discovered proteomic changes that are unique to SPN compared to other pancreatic neoplasms and benign tissue (Fig. 2A, B, C, D, E). These unique proteomic changes in SPN mapped to biological processes such as lysosomal or small vesicle-related processes (Fig. 2F), which was also highlighted by protein-protein MCODE network analyses (Fig. 2G). This is consistent with our finding that MITF and TFE3, upstream transcription factors of lysosome-related genes [48], are overexpressed in SPN, but not in four other pancreatic tumor types (PDAC, PanNET, ACC, and PBL) or benign pancreatic tissues (Fig. 3A, B, C). Consistent with this observation, SPN cells are often characterized by cytoplasmic hyaline globules that resemble complex secondary lysosomes. Interestingly, we discovered that the specificity of MITF for SPN was higher than that of TFE3, a finding that has not previously been described. While TFE3 IHC has been considered a specific diagnostic protein assessment for SPN [58, 59], recent studies have reported TFE3 expression in other pancreatic tumors, with positivity rates of up to 14.3% in PDAC and 23.5% in PanNET, significantly limiting its diagnostic specificity. This limitation underscores the need for more specific biomarkers. Our proteomic and IHC data show that MITF is strongly and specifically overexpressed in SPN, but not in PDAC, PanNET, or benign pancreatic tissues (Fig. 3A, B, C, D). To further validate this finding, we expanded our IHC analysis to include additional SPN, PDAC, and PanNET cases, confirming the high specificity of MITF expression for SPN (Fig. 3C). These results suggest that MITF may serve as a more reliable and diagnostically useful marker for SPN than TFE3, particularly in the differential diagnosis of pancreatic neoplasms with overlapping histologic features.

Although the functional roles of MITF/TFE3 in SPN are not clear at this point, MITF/TFE family transcription factors increased lysosomal breakdown in PDAC cell lines and provided tumor cells with intracellular and extracellular nutrients [60, 61], suggesting that MITF/TFE3 may have similar roles in SPN for survival in low-nutrient environments. MITF/TFE3 have been reported to sense nutrition deprivation and drive metabolic adaptation [49, 50, 62]. Proteome-based metabolic pathway analyses may offer insights into the distinct biology of SPN in comparison to PDAC and PanNET. SPNs exhibit a metabolic profile that more closely resembles benign pancreatic tissue, in contrast to the highly glycolytic and aggressively proliferative phenotype of PDAC (Fig. 3E). Notably, fatty acid β-oxidation is prominent in SPNs, whereas PDAC is characterized by a downregulated TCA cycle and enhanced glycolysis, indicative of the Warburg effect. This is consistent with the clinical observation that SPNs represent relatively indolent tumors that favor oxidative metabolism over rapid glycolytic flux. Despite their more “benign-like” oxidative metabolism profile, SPNs demonstrate elevated levels of proteasome activation, highlighting the tumor’s requirement for effective protein quality control and turnover (Fig. 3F). Tumor cells frequently accumulate misfolded or excess proteins due to stress from nutrient fluctuations, intracellular pH changes, and oncogenic drivers [63–65]. The increased proteasomal activity in SPNs likely reduces protein aggregation stress, thereby maintaining proteostasis and supporting tumor survival under nutrient deprivation. These findings are consistent with the nuclear expression of TFE3 in SPNs, as TFE family transcription factors regulate lysosomal and autophagy-related genes, as well as proteasome-associated factors [49, 50, 62]. Elevated MITF/TFE3 activity may orchestrate a metabolic and catabolic environment that provides advantages in challenging microenvironments, where reliance on fatty acid oxidation necessitates precise regulation of cellular components and redox balance. The results suggest that SPNs occupy a unique metabolic state: reminiscent of benign pancreatic tissue in their oxidative metabolism, yet exhibiting significantly elevated proteasome activation typical of some cancers [66, 67]. Future research may explore how SPN utilizes fatty acid oxidation with enhanced proteasome activity to drive tumor growth and survival, and whether these characteristics could be exploited for targeted therapeutic interventions [67].

Our study is the first to describe the tumor immune microenvironment profile of SPN at the proteome level (Fig. 4). The comparative analysis of SPN, PanNET, and PDAC revealed distinct tumor immune microenvironments. xCell-based deconvolution determined that SPN is relatively “immune cold,” with low T and B lymphocyte infiltration but abundant M2-type macrophages and myeloid cells, creating an immunosuppressive tumor microenvironment. Consistent with this data, microscopic examination of SPN shows frequently aggregates of foamy macrophages. PDAC exhibited an “immune hot” phenotype, with enhanced T-cell infiltration, robust MHC class I activity, and elevated IFNγ pathway signaling, though its dense stroma poses therapeutic challenges for immunotherapies [68, 69]. SPNs reduced JAK/STAT and IRF-mediated signaling, together with low expression of antigen processing machinery (APM) proteins, likely contributes to suboptimal MHC class I presentation and limited effector T-cell engagement. These findings suggest that SPNs immune-cold environment may pose significant hurdles to the development of successful immunotherapeutic approaches. Strategies that enhance APM component expression or shift macrophage polarization away from the M2 phenotype may transform SPNs immune-cold environment into a more activated state [70, 71].

We also used our deep proteomic data to characterize RTK signaling pathway activation in SPN. Targeting RTK pathways is an effective treatment strategy for various tumor types [53–56]. Our data showed that three RTK pathways (PDGF, FGFR, and ERBB) are significantly activated in SPN based on proteomic gene set enrichment (Fig. 5A). In addition, more than 50% of SPNs exhibited positivity for PDGFRA, ERBB2 (HER2), FGFR1, or FGFR4 protein by IHC assessment (Fig. 5B). These findings are potentially noteworthy, as no targeted drug therapies are currently available for SPN. 8–13% of SPN cases show distant metastasis or recurrence [5]. Systemic targeted therapy options against these RTKs, which are currently not in use for SPN, may greatly benefit patients with metastatic or recurrent SPN.

Our study has certain limitations. Although our cohort is larger than those in previous studies, it remains relatively modest in size. The analyses are based on bulk FFPE proteomics. Currently, there are no validated SPN models available such as cell lines or organoids. Future research should focus on multi-center validation of MITF using diagnostic metrics, the development of SPN cell/organoid/PDX models for mechanistic testing (including MITF, lysosome-related autophagy, AMPK/mTORC signaling, and RTKs), and the expansion of phosphoproteomics or spatial proteomics to enhance pathway and immune insights.

Proteomic profiling of SPN identifies potentially targetable proteins and pathways. While further validation is necessary, these findings represent a promising step towards the development of molecularly targeted therapies for SPN.

## Conclusions

In summary, we described the proteomic characteristics of SPN, including enriched pathways, diagnostic protein markers, unique metabolic adaptation processes, immune microenvironment, and possible therapeutic targets. Our results provide a unique proteomic contribution to the understanding of SPN biology and highlight differences to other pancreatic tumors.

## Electronic supplementary material

Below is the link to the electronic supplementary material.

Supplementary Material 1

Supplementary Material 2

Supplementary Material 3

## Data Availability

The mass spectrometry proteomics data from this study can be found in the PRIDE repository [72] with the ID PXD039182.

## Abbreviations

PDAC: Pancreatic ductal adenocarcinoma
SPN: Solid pseudopapillary neoplasm
PanNET: Pancreatic neuroendocrine tumor
